# Endocrown: A Conservative Approach in the Management of Endodontically Treated Teeth

**DOI:** 10.7759/cureus.60686

**Published:** 2024-05-20

**Authors:** Saee Wazurkar, Aditya Patel, Joyeeta Mahapatra, Mrinal Nadgouda, Lalit Pawar

**Affiliations:** 1 Department of Conservative Dentistry and Endodontics, Sharad Pawar Dental College and Hospital, Datta Meghe Institute of Higher Education and Research, Wardha, IND

**Keywords:** endodontically treated teeth, cad cam milled zirconia prosthesis, cad-cam, zirconia, endocrown

## Abstract

The outcome of an endodontic procedure determines the clinical success of the treated tooth. A post-endodontic restoration will restore the tooth's form, function, and aesthetics while preserving and safeguarding its existing tooth structure. To restore endodontically treated teeth with the best possible tissue preservation, the least invasive preparation is the aim. Full-coverage crowns are still more popular than partial-coverage crowns. Conservative dental procedures such as inlays, overlays, and endocrowns maximize the amount of tooth structure that is intact while minimizing the amount of tooth structure that is removed. Compared to posts, cores, and crowns, endocrowns offer several advantages in terms of ease of preparation, application, and reduced clinical visits and time. Endocrown is a simple, minimally invasive preparation usually given when margins are supragingival, which makes it self-cleansable and maintains natural tooth contact, preventing interference with periodontal tissue. This case report focuses on managing endodontically treated teeth with the fabrication of endocrown using computer-aided design (CAD) and computer-assisted manufacturing (CAM) techniques.

## Introduction

Root canal treatment is a normal dental procedure, but it can eventually weaken the tooth structure [1]. This loss of structure can reduce the tooth's mechanical properties and fracture resistance, affecting its overall prognosis [2]. Loss of tooth structure can result from trauma, tooth decay, or while deroofing the access cavity [1].

The type of restorative material used, along with proper restoration, conserves the integrity of the tooth and also increases its longevity [3]. Final post-endodontic restoration depends upon the amount of tooth structure left and the type of tooth, whether it is anterior or posterior. An anterior tooth with a limited access cavity can be filled directly with any adhesive cement [4]. Dealing with posterior teeth cuspal coverage is mandatory to maintain its anatomical form and to resist the high masticatory load [5]. A tooth with considerable tooth loss may require a post and core for added retention, but an intracanal post can weaken the tooth structure, leading to tooth fracture [6].

With the emphasis on minimally invasive concepts and progress made in adhesive dentistry, endocrown restorations have been introduced as an alternative option for rehabilitating endodontically treated teeth [7]. Bindl and Mörmann coined the term "endocrown" in 1999. They used the pulp-chamber walls' micromechanical retention qualities to describe an adhesive monolithic ceramic restoration that had been placed in the pulp chamber [8]. This paper aims to present a clinical case of endodontic treatment of a mandibular molar that was restored with an aesthetic and conservative posterior endocrown.

## Case presentation

The primary concern of a 38-year-old male patient who came to the Department of Conservative Dentistry and Endodontics was that he had difficulty during mastication due to the coronal destruction and dislodgment of the previous restoration in the lower left back region of the jaw for the past 15 days. The patient was initially in good health when he started experiencing difficulty in mastication due to a dislodged prosthesis. The patient underwent endodontic treatment with the same tooth six months ago. The past medical history was nonsignificant for the current clinical situation, and a radiographical examination shows a dislodged prosthesis with tooth number 36. The tooth was non-tender on percussion, and there was no history of pain or signs of swelling with the concerned tooth. On radiographic examination, a well-executed root canal filling was seen in tooth 36.

Endocrown preparation

**Figure 1:**
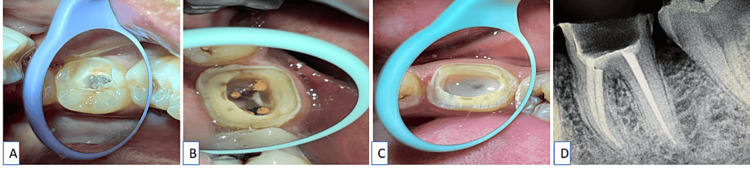
(A) Previous root canal treatment on tooth number 36; (B) tooth preparation performed on tooth number 36; (C) glass ionomer cement to seal the pulpal floor; (D) radiograph taken following tooth preparation with tooth number 36

The method suggested by Bindl and Mörmann for tooth preparation was followed [8]. Figure 1A shows the dislodged restoration concerning tooth number 36. For the endocrown preparation, cervical margins were shaped into a chamfer contour, but the residual coronal tooth structure was left with a constant thickness of 1.5 mm. This was accomplished with a high-speed diamond bur SO-21 (produced by Dia-Burs, Mani, Tochigi, Japan) with constant cooling (Figure 1B). The cervical margin was kept supragingival, and enamel walls smaller than 2 mm were removed. The occlusal reduction was carried out using a diamond wheel bur (WR-13, Dia-Burs, Mani, Tochigi, Japan). Because of its specific shape, a diamond wheel bur was used to ensure an even surface. A consistent transition between the access cavity and the prepared coronal pulp chamber was ensured by carefully maintaining an occlusal convergence ranging between 7° and 10° while aligning the bur parallel to the tooth's long axis.

**Figure 2:**
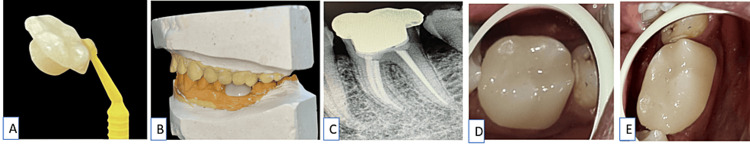
(A) CAD/CAM-processed and fabricated zirconia endocrown; (B) occlusal fit check of the endocrown on the cast; (C) radiograph following the cementation of the endocrown with tooth number 36; (D) clinical photograph showing the cemented endocrown with tooth number 36; (E) clinical photograph after the six-month follow-up CAD: computer-aided design; CAM: computer-aided manufacturing

After the pulp chamber and coronal orifices were completely sealed with a glass ionomer (Type II) restorative cement (GC Corporation, Tokyo, Japan), as shown in Figure 1C, the gutta-percha was removed up to a depth of 2 mm below the level of each orifice, that is, from the mesiobuccal, mesiolingual, and distal canals. Figure 1D shows a radiograph after tooth preparation. Subsequently, the impression was made using polyvinylsiloxane material through a step dual-viscosity technique (Reprosil LV Tube, Aquasil Soft Putty, Dentsply Sirona Inc., Charlotte, North Carolina), following a detailed evaluation of the complete cavity and occlusal area. The cast was then fabricated, as illustrated in Figure 2A. In the present case study, an interim prosthesis was made during the interappointment period, and an interim prosthesis was fabricated using self-curing resin.

Zirconia (Figure 2B) shows the occlusal fit of a computer-aided design (CAD). A computer-assisted manufacturing (CAM) endocrown was fabricated using Cercon (Dentsply Sirona Inc.), which is available in a disc shape. The removal of interim restoration was uneventful. Every tooth surface was cleaned meticulously. Accurate marginal adaptation mesiodistal dimension, proper shape in conjunction with surrounding tissues, color resemblance to natural adjacent teeth, and adequate aesthetics were all checked. Factors such as occlusion and contact points were also evaluated and altered as needed.

Etching (Prime Dental, Thane, India) was performed for 30 seconds on the enamel and 15 seconds on the dentinal surface. After thoroughly cleaning and drying, a layer of adhesive was applied to the tooth-coated surface and then light-cured for 20 seconds. Once the prosthetic endocrown was placed within the tooth and covered with a small amount of dual-polymerizing resin, polymerization was carried out every five seconds. This technique enables the removal of the extra cement. It was then light-cured from all surfaces for 60 seconds. Figure 2C shows the post-endocrown cementation radiograph. The prosthesis was inspected with ceramic finishing instruments for any interocclusal disturbances (Figure 2D). The patient was re-evaluated in a six-month follow-up (Figure 2E). The clinical examination revealed that the endocrown functioned normally without any symptoms, and there was no sign of abrasion to the patient's teeth.

## Discussion

The most effective strategy to follow for restoring root canal-treated teeth has long been debated in the literature. Preserving the remaining healthy dental tissues is essential for long-term success because they are anchors for tooth-restoration complexes [9]. Endocrowns are found to be valuable for the loss of coronal tooth structure as they conservatively prepare the tooth structure [10]. Endocrowns are recommended for teeth with calcified canals, short clinical crowns, and a loss of coronal tooth structure.

Nevertheless, an additional retentive mechanism must be added if the tooth structure that remains is inadequate to hold the core in place. Typically, a post or dowel is inserted to preserve the main structure; these posts can be one-piece custom-made posts and cores or prefabricated posts with a direct core [5]. Previous research has demonstrated that the post merely helps with the retention of the restoration, contrary to earlier theories that the post and core reinforced the remaining tooth structure [11]. Conversely, removing the radicular structure to place the post could weaken the root and increase its vulnerability to fracture [12]. The development of dentin bonding agents marked a turning point in the restoration of teeth treated with endodontia; this led to a decrease in the preference for radicular post-insertion, provided that there is adequate surface area available for micromechanical retention [13]. In this study, to enhance the surface roughness and increase the bondability of the material, sandblasting was performed [14]. Recently, a new type of light-cured adhesive material has been developed that includes primers containing 10-methacryloyloxydecyl dihydrogen phosphate (MDP) monomer that could be used in combination with micromechanical retention to strengthen chemical bonds [15].

In 1995, Pissis introduced a novel approach that combined the porcelain core and crown into a single unit, termed the "monobloc technique," as an alternative to the conventional metal post and core method. This innovation aimed to enhance dental restoration [16]. Subsequently, in 2018, Dartora et al. investigated the biomechanical response of teeth treated with endodontic therapy and restored with various endocrown extensions within the pulp chamber. Their findings suggested that greater endocrown extensions resulted in improved mechanical performance. Specifically, compared to a 1 mm extension, which exhibited reduced fracture resistance and increased potential for rotation during function, a 5 mm extension demonstrated decreased stress intensity and a more favorable distribution pattern [17,18].

Bindl and Mörmann introduced endocrown in 1999 as it requires minimal preparation, which provides structural stability and durability as it conservatively prepares the tooth [8]. The endocrown displays superior anatomic contour and surface textures made using the CAD/CAM technique compared to those fabricated by lab technicians [19]. The success of endocrown restorations made with CEREC 3 and Vita Mark II feldspathic ceramic in a CAD-CAM system over the past 12 years has been the subject of recent scientific studies. Of 55 patients, the estimated success rate for molars is 90.5%, and for premolars, it is 75% [20].

The outcomes of studies on the clinical survival and in vitro fracture strength of endocrown restorations were thoroughly compared with those of more conventional options, such as intraradicular posts, direct composite resin, or inlay/onlay restorations, by Sedrez-Porto et al. Their findings indicate that endocrowns function either as well as or better than conventional therapies [21]. Furthermore, Schultheis et al. suggest that end crowns, which may reduce fracture failures when arranged in a bilayer configuration, are more reliable for posterior teeth supporting significant loads [22].

Belleflamme et al. propose that endocrowns might present a feasible solution for extensively damaged molars and premolars. This applies particularly in cases with significant crown tissue loss, occlusal risk factors, or challenging occlusal relationships [23].

## Conclusions

Endocrowns are a recently developed method that is highly valued for their ease of use and numerous advantages, including the reduction of interfaces in a restorative system. The unique aspect of the preparation design lies in its conservative and constrained approach to biological width. However, it is crucial to remember that there are potential risks, such as root fracture and debonding in teeth with endodontic treatment, due to the fact that the harder ceramic has a lower modulus of elasticity than dentin. For long-term success, careful case selection is therefore essential. The longevity and success of endocrowns depend on several factors, which include bonding agent selection, proper ceramic material selection, case selection, and appropriate preparation techniques.

## References

[1] Ciobanu P, Manziuc MM, Buduru SD, Dudea D (2023). Endocrowns - a literature review. Med Pharm Rep.

[2] Nayak M, Jose A (2020). Endocrown: an option for rehabilitation of badly mutilated tooth: a case report. Int J Appl Dent Sci.

[3] Ferrari M, Vichi A, Mannocci F, Mason PN (2001). Retrospective study of the clinical performance of fiber posts. Am J Dent.

[4] Morgano SM (1996). Restoration of pulpless teeth: application of traditional principles in present and future contexts. J Prosthet Dent.

[5] Elagra M (2019). Endocrown preparation: review. Int J Appl Dent Sci.

[6] Dogui H, Abdelmalek F, Amor A, Douki N (2018). Endocrown: an alternative approach for restoring endodontically treated molars with large coronal destruction. Case Rep Dent.

[7] Magne P (2012). Pascal Magne: 'it should not be about aesthetics but tooth-conserving dentistry'. Br Dent J.

[8] Bindl A, Mörmann WH (1999). Clinical evaluation of adhesively placed Cerec endo-crowns after 2 years - preliminary results. J Adhes Dent.

[9] Chang CY, Kuo JS, Lin YS, Chang YH (2009). Fracture resistance and failure modes of CEREC endo-crowns and conventional post and core-supported CEREC crowns. J Dent Sci.

[10] Bindl A, Richter B, Mörmann WH (2005). Survival of ceramic computer-aided design/manufacturing crowns bonded to preparations with reduced macroretention geometry. Int J Prosthodont.

[11] Sevimli G, Cengiz S, Oruc MS (2015). Endocrowns: review. J Istanb Univ Fac Dent.

[12] Garhnayak L, Parkash H, Sehgal DK, Jain V, Garhnayak M (2011). A comparative study of the stress distribution in different endodontic post-retained teeth with and without ferrule design—a finite element analysis. ISRN Dent.

[13] Fages M, Bennasar B (2013). The endocrown: a different type of all-ceramic reconstruction for molars. J Can Dent Assoc.

[14] Muhammed HA, Mahmoud EM, Fahmy AE, Nasr DM (2023). The effect of sandblasting versus acid etching on the surface roughness and biaxial flexural strength of CAD/CAM resin-matrix ceramics (in vitro study). BMC Oral Health.

[15] Anh NV, Son TM, Ngoc VT, Ha PT, Hung DT, Nga MH, Tra NT (2023). Shear bond strength of MDP-containing light-cured veneer adhesive system to zirconia with different surface preparations. J Adhes Sci Technol.

[16] Pissis P (1995). Fabrication of a metal-free ceramic restoration utilizing the monobloc technique. Pract Periodontics Aesthet Dent.

[17] Dartora NR, de Conto Ferreira MB, Moris IC (2018). Effect of intracoronal depth of teeth restored with endocrowns on fracture resistance: in vitro and 3-dimensional finite element analysis. J Endod.

[18] Taha D, Spintzyk S, Schille C, Sabet A, Wahsh M, Salah T, Geis-Gerstorfer J (2018). Fracture resistance and failure modes of polymer infiltrated ceramic endocrown restorations with variations in margin design and occlusal thickness. J Prosthodont Res.

[19] Litzenburger AP, Hickel R, Richter MJ, Mehl AC, Probst FA (2013). Fully automatic CAD design of the occlusal morphology of partial crowns compared to dental technicians' design. Clin Oral Investig.

[20] Bencun M, Ender A, Wiedemeier DB, Mehl A (2020). Fracture load of CAD/CAM feldspathic crowns influenced by abutment material. Materials (Basel).

[21] Sedrez-Porto JA, Rosa WL, da Silva AF, Münchow EA, Pereira-Cenci T (2016). Endocrown restorations: a systematic review and meta-analysis. J Dent.

[22] Schultheis S, Strub JR, Gerds TA, Guess PC (2013). Monolithic and bi-layer CAD/CAM lithium-disilicate versus metal-ceramic fixed dental prostheses: comparison of fracture loads and failure modes after fatigue. Clin Oral Investig.

[23] Belleflamme MM, Geerts SO, Louwette MM, Grenade CF, Vanheusden AJ, Mainjot AK (2017). No post-no core approach to restore severely damaged posterior teeth: an up to 10-year retrospective study of documented endocrown cases. J Dent.

